# Supporting IOL'S in a Deficient Capsular Environment: The Tale of No “Tails”

**DOI:** 10.1155/2021/9933486

**Published:** 2021-09-13

**Authors:** Domenico Boccuzzi, Date Purva, Vincenzo Orfeo, Pasquale Napolitano, Alessandro Mularoni, Aurelio Imburgia, Matteo Forlini

**Affiliations:** ^1^Ophthalmology Unit “Clinica Mediterranea”, Naples, Italy; ^2^Valvekar Medical & Research Centre, Solapur, India; ^3^Department of Medicine and Health Sciences “V. Tiberio”, University of Molise, Campobasso, Italy; ^4^Department of Ophthalmology, San Marino State Hospital, San Marino, Italy

## Abstract

**Purpose:**

To evaluate the efficacy and safety of the following three distinct surgical procedures for secondary IOL implantation without capsular support: Iris-claw lens, flanged transscleral fixated IOLs (Yamane technique), and sutureless transscleral hook IOL fixation (Carlevale IOL).

**Materials and Methods:**

In this retrospective comparative study, three different sutureless IOL implantation techniques were compared in patients without any capsular support. Visual acuity and outcomes were analyzed in 24 eyes of 23 patients (14 male and 9 female). Study included 13 iris-claw lenses (Artisan Ophtec), 6 flanged transscleral fixated IOLs (Yamane technique using a MA60MA Alcon Inc IOL), and 5 transscleral Carlevale IOLS (Carlevale IOL, Soleko, Italy).

**Results:**

logMAR mean best-corrected visual acuity (BCVA) improved from 0.49 ± 0.19 to 0.19 ± 0.10 at three months after surgery (*p* < 0.05). Postoperative BCVA was similar in all three groups, and no intergroup difference was noted. Three eyes (12.5%) had a raised IOP >25 mmHg, 2 eyes (8%) presented a subluxated/dislocated IOL, 4 eyes (16%) had corneal edema longer than 7 days, 3 eyes (12.5%) had irregular pupil profile, 2 eyes (8%) had vitreous hemorrhage, 7 eyes had (29%) corneal astigmatism over 3 diopters, and one patient (4%) developed cystoid macular edema (CME).

**Conclusions:**

All three surgical procedures can be considered adequate to correct aphakia in patients without capsular support with significant improvement in visual acuity and low complication.

## 1. Introduction

Modern cataract surgery has excellent results and a rapid visual recovery after intraocular lens (IOL) implantation in the capsular bag (PC-IOL). However, lens dislocation in the vitreous cavity, posttraumatic cataract surgery, pseudoexfoliation (PXF), Marfan syndrome, and Ehlers Danlos syndrome may encounter an issue of inadequate capsular support not suitable for in-the-bag or ciliary sulcus IOL implantation [1].

Sutured transscleral IOL fixation of three-piece posterior chamber IOLs is a valid procedure for these patients. The downside of this procedure is longer surgical time and a higher complication rate [2–8]. Hence, there is a need for a simpler procedure with the lower complication rate and faster functional recovery.

Various procedures such as iris-claw, flanged transscleral fixated IOLs (Yamane technique), and sutureless transscleral hook IOL fixation (Carlevale IOL) have been described for the considered indications.

This comparative retrospective study aims to evaluate and compare these three surgical procedures with respect to their outcomes and complications.

## 2. Materials and Methods

This is a nonrandomized comparative retrospective study carried out on 23 patients (24 eyes). The study was conducted between January 2017 and December 2018 at Clinica Mediterranea, Naples, Italy. Relevant data of the study population were drawn from informatics medical records. All subjects had lens-related issues with an inadequate capsular support and needed IOL implantation. All patients were informed about the risks and benefits of the surgery, and a written informed consent was obtained. The study was conducted in accordance with the tenets of the Helsinki Declaration.

Preoperative and postoperative ophthalmic evaluation included Snellen BCVA, slit lamp examination, Goldman applanation tonometry for intraocular pressure (IOP) measurement, and a detailed fundus examination. All surgical procedures were performed under complete aseptic precautions. Surgeries were performed under peribulbar anesthesia (ropivacaine hydrochloride 10 mg/ml).

Low intraocular pressure was considered at IOP <6 mm Hg, while a reading of more than 25 mm Hg was considered as high. Biometry was performed in all patients, including the pseudophakic eyes, and IOL power was calculated with Haigis, SRK-T, Holladay 1 e Hoffer Q formulas. We defined primary surgery as the first surgical intervention (PHACO, femtolaser-assisted capsular surgery, FLACS, or intracapsular extraction (ICCE). Late onset subluxation secondary to PXF was also included here. Secondary surgery was defined as the surgical approach necessary for extraction of subluxated/dislocated IOL or residual lens remnants along with secondary IOL implantation (anterior vitrectomy (AV) or PPV). The documentation included the type of surgical procedure for cataract remnants removal, surgical procedure for IOL implantation, perioperative complications, and outcomes at the end of the 6 months follow-up period.

### 2.1. Surgical Technique

Cataract surgery was performed using a phacomachine with a combined torsional and longitudinal US system (Centurion Vision System, Alcon, Fort Worth, Texas, USA.). In aphakic patients without nuclear fragments in the vitreous, the anterior vitrectomy was performed with a 23G vitrector (Centurion Vision System, Alcon, Fort Worth, Texas, USA).

A 23G pars plana vitrectomy (PPV) (Constellation Vision System, Alcon, Fort Worth, Texas, USA.) was done when nuclear fragments or IOLS were dislocated in the vitreous cavity. Nuclear fragments were removed after core vitrectomy, inducing posterior vitreous detachment and vitreous base shaving [9]. Perfluorocarbonate liquid (PFCL) was injected for macular and posterior pole protection. Nuclear fragments were removed using the phacofragmatome or vitrectomy cutter. When an IOL subluxation occurred with the bag itself, a posterior vitrectomy was performed to release the vitreous adhesions with the bag-IOL complex. The lens itself was brought into the anterior chamber using vitreous forceps.

### 2.2. Iris-Claw Surgical Technique

Artisan (Ophtec, Groningen, Netherlands) iris-claw lens was used wherever indicated. This is a 5 mm biconvex PMMA lens with a greater diameter of 8.5 mm. IOL power was calculated using SRK/T formula. The constant for the correct IOL power calculation in the anterior chamber (over the iris) was 115.0.

A superior 5 mm clear cornea incision with two side ports 180° apart was performed. After a thorough anterior/posterior vitrectomy, miosis was achieved by injecting acetylcholine chloride in the anterior chamber (Miovisin, Farmigea, 2 mg/2 ml). Sodium hyaluronate 1.4% (Healon GV, Johnson & Johnson Vision) was injected for maintenance of the anterior chamber endothelial protection and to facilitate IOL handling. The IOL was inserted in the anterior chamber in a vertical position to take advantage of the smaller diameter of the lens and then was rotated by 90° for the correct position and enclavation on the iris. The lens was held with Buratto's forceps for the enclavation procedure. The IOL was enclaved using a needle through a lateral paracentesis side port. At the end of the procedure, a small iridectomy was performed to avoid pupillary block. The main incision was sutured with four interrupted 10/0 nylon sutures. Healon was washed out from the anterior chamber, and 1 mg of cefuroxime (Aprokam, Theà) was injected in the anterior chamber [10] (Supplementary Video 1: https://drive.google.com/file/d/1a6SridVD9ZBNOmdvftMtTeK-NH3-nf7Y/view?usp=sharing).

### 2.3. Transscleral Implantation Technique

The three-piece IOL, when present, was unleashed from the capsular bag and prepared to be implanted using the transscleral technique. One of the haptics was extruded from the main incision to avoid a subluxation of the lens itself. This sutureless intrascleral three-piece IOL fixation is a technique also known as flanged IOL fixation and was described by Yamane et al. in [11]. When a single-piece IOL was present, the lens was cut with scissors and explanted from the main incision itself. Three-piece IOL (MA60MA, Alcon Inc.) was injected in the anterior chamber leaving the trailing haptic out of the main incision to avoid the lens drop in the vitreous cavity. An angled sclerotomy was made through the conjunctiva using a 30-gauge thin-wall needle (TSK ultrathin-wall needle, Tochigi Seiko, Tochigi, Japan) at 2 mm from the limbus. The leading haptic was threaded into the lumen of the needle using forceps. A second sclerotomy then was made with a 30-gauge thin-wall needle that was 180° from the first sclerotomy. The trailing haptic was inserted into the lumen of the second needle, while the first needle was put on the eye lid. Both haptics were externalized onto the conjunctiva using the double-needle technique. The ends of the haptics were cauterized using an ophthalmic cautery device (Accu-Temp Cautery, Beaver Visitec, Waltham, MA) to make a flange with a diameter of 0.3 mm. The flange of the haptics was pushed back and fixed into the scleral tunnel. A peripheral iridotomy was performed using the vitrectomy cutter after miosis to avoid iris capture of the IOL. At the end of surgery, 1 mg of cefuroxime (Aprokam, Theà) was injected in the anterior chamber.

### 2.4. Carlevale's Lens Implantation Technique

Carlevale's lens is a single piece, 25% water hydrophilic acrylic IOL, with a 6.5 mm optic, a 13.5 mm diameter, and 10° vaulted haptic with a retina vault and retina convexity. Carlevale IOL has a correct direction of the implant, indicated by the presence of two small tags on the haptics and a harpoon for sutureless transscleral fixation. IOL's power range is between −5 and +35 diopters, and the A constant is 118.5. After corneal white to white diameter evaluation (WTW), the infusion line is positioned at inferotemporal quadrant. A limited conjunctival peritomy, 2 partial 4 × 4 mm thickness scleral flaps were made and hinged at the limbus 180° apart. Then, two sclerotomies using a 25-gauge needle were placed at 1.5–2.0 mm from the limbus in correspondence to the three and nine o clock position (Supplementary video 2: https://drive.google.com/file/d/1uR_B8y4j0Nwi3LerWOMcoCmwGam7-ktU/view?usp=sharing). The Carlevale IOL was injected into the anterior chamber through a corneal tunnel using a Viscojet injector (Medical Viscojet 2.2 mm), and the leading plug was grasped with crocodile tip forceps inserted into the vitreous chamber through the sclerotomy and then externalized under the scleral flap in a single maneuver. Then, the trailing plug was grasped and externalized with 2 forceps using the handshake technique; IOL centration was achieved without performing extra-intraoperative maneuvers. Scleral flaps and conjunctival wound were sealed with nylon 10/0 and polyglactin 8/0 (Vicryl), respectively. A 10/0 nylon stitch is positioned on the main incision suture, and 1 mg cefuroxime is injected in the anterior chamber [12, 13].

### 2.5. Statistical Analysis

Statistical analysis was performed using SPSS software (version 26, IBM Corp.) A paired *t*-test was used to compare preop and postop visual acuity of the three groups. A *p* value of less than 0.05 was considered statistically significant. Visual acuity was converted to a logarithm of the minimum angle of resolution (logMAR) for analysis. The ANOVA analysis followed by the Bonferroni test was used to compare postop visual acuity between the three groups.

## 3. Results

We have compared postop visual acuity and complications of three different sutureless IOL implantation techniques in patients without any capsular support. Out of 23 patients, 14 were male (60%) and 9 were female (40%).

We used three implantation techniques for this cohort of patients: in group 1 (13/24 eyes, 54.1%), iris-claw lenses were implanted in the anterior chamber (Artisan Ophtec). In group 2 (6/24 eyes, 25%), sutureless intrascleral three-piece IOL (MA60MA, Alcon Inc.) was used. In group 3 (5 out of 24 eyes, 20.8%), transscleral IOL fixation with an intrascleral plug using Carlevale's IOL (Carlevale IOL, Soleko, Italy) placement was done. Various etiological causes of insufficient capsular support with the type of different IOLs are given in Table 1.

Table 2 provides the etiology of loss of capsular support, surgery type (kind of vitrectomy).

Ten out of 24 eyes (41.6%) had a posterior capsular rent (PCR) during cataract surgery. Out of 10 PCR cases, 6 eyes (25%) required PPV to remove cataract remnants dislocated in the vitreous cavity, while 4 eyes (16%) required only anterior vitrectomy. Out of 10 PCR cases, 6 eyes had PXF with zonular deficiency. Out of 6 PXF cases, 1 case was planned for intracapsular cataract extraction (ICCE) with vitrectomy due to evident phacodonesis in more than 270°. Three cases required a posterior approach to complete the vitrectomy, and 3 cases were managed with anterior vitrectomy alone.

**Figure 1:**
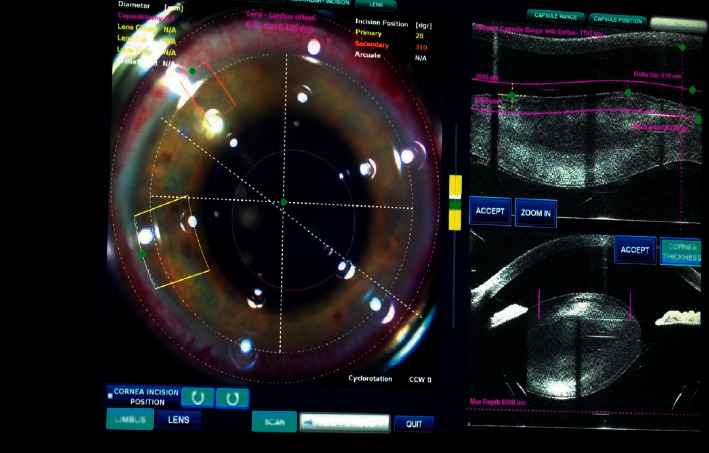
Femtolaser-assisted cataract surgery in Marfan's syndrome demonstrating zonular disinsertion with nucleus subluxation.

Three eyes (12.5%) had IOL-bag complex subluxation due to PXF. All cases needed 3 ports PPV with IOL removal. In 2 eyes, the subsequent same three-piece IOL was used for transscleral IOL fixation. In the third eye, an iris-claw was implanted. In 3 cases (12.5%) with Marfan's-associated subluxation, 2 eyes underwent PHACO with anterior vitrectomy with iris-claw implantation. In 1 case, FLACS (femtolaser-assisted cataract surgery) was used for capsulorrhexis and nucleus fragmentation where Carlevale's lens were implanted (Figure 1). Two patients (8%) had a traumatic subluxated cataract that required PPV with Carlevale's lens implantation.

Patients were followed up at postop day one and then at one week and one month. The last follow-up was at six months.

Improvement in mean visual acuity (BCVA) is given in (Table 3).

BCVA comparison evaluated with ANOVA followed by Bonferroni's test didn't show any statistical significance (significant *p* > 0.05) related to the different surgical techniques used (Table 4).

### 3.1. Complications

Some complications were noted after primary surgery, but none resulted in diminished visual acuity. Complications related to the lens implanted are given in Table 5. Raised IOP was noted in 4 eyes (16%). One patient (total 8%) in the transscleral flanged group and in the iris-claw group had IOL malposition and subluxation. The one in the iris-claw group required secondary surgery because of the loss of hooking on the iris.

Four of the 24 eyes (16%) had corneal edema secondary to raised IOP, which lasted over 7 days (two in the transscleral flanged group and two in the iris-claw group). It resolved with topical antiglaucoma therapy.

Cystoid macular edema (CME) occurred in just one eye (4%) in the iris-claw group that required NSAID eye drops. The CME resolved after one month of topic therapy.

Three eyes (12.5%) showed pupillary anomalies in the iris-claw group, related to improper iris hooking. None required IOL repositioning. In two eyes (one each in the transscleral and Carlevale's group), vitreous hemorrhage (VH) occurred possibly following near-to-limbus sclerotomy. Seven eyes (all from iris-claw group) developed high postoperative astigmatism (>3D). This was tackled by sequential removal of the main incision sutures over a period of time. Yet, one patient had persistent astigmatism >3D until final follow-up. There was no incidence of retinal or choroidal detachment or endophthalmitis.

## 4. Discussion

Aphakia with inadequate capsular support can be seen in several conditions such as postcomplicated cataract surgery, PXF syndrome, subluxation secondary to capsular instability in Marfan's syndrome, Ehlers Danlos, or in traumatic cataract. These conditions require an individualized approach with different IOL implantation techniques. Angle-supported, scleral or iris-supported IOLs are used, each having their pros and cons. The main purpose of the study was to evaluate three different surgical techniques and compare their efficacy and complications.

Secondary IOL implantation can be divided into sutured and sutureless techniques. Sutured surgical techniques require a 10/0 or 9-0 Prolene suture to secure the IOL to the scleral tissue. Sutured techniques are associated with complications such as suture breaks (Prolene), conjunctival erosion, cheese wiring, and rarely secondary retinal detachment [14–16]. Hence, sutureless techniques were the need of the hour (2–8).

Gabor and Pavilidis first described a new sutureless technique in which the haptics were extruded out by a sclerotomy and tucked in a scleral tunnel prepared ad hoc [17, 18]. A faster glued IOL technique was described by Agarwal et al., wherein he used fibrin glue to fix the scleral flaps. [19]. Both the Scharioth and Agarwal sutureless surgical procedures are prone to postoperative hypotony [18, 19].

In 2017, Yamane et al. proposed the flanged intrascleral IOL fixation, which could be considered the optimization of both the Schariot and Agarwal techniques [11]. This double-needle technique entailed externalization of two haptics using a 30-gauge thin-wall needle (TSK ultrathin-wall needle; Tochigi Seiko, Tochigi, Japan) at 2 mm from the limbus. This not only provided guidance for extrusion of the haptics but also eliminated the need of peritomy and scleral flap creation. This technique avoids all complications related to sutures. The small size of the tunnel incision reduces the risk of iris prolapse, leakage, anterior chamber shallowing, and suprachoroidal hemorrhage. Suture less techniques are quicker with a shorter rehabilitation period. It also allows salvage of the previously implanted three-piece IOL. We have also been able to do the same in one of our patients. This technique may require a fair learning curve so as to avoid tilt or decentration [20–22].

Worst et al. (in 1972) original iris-claw lens has been modified over time (Artisan (Ophtec)) to avoid corneal decompensation. [23] Verisyse (2005, AMO, presently Johnson & Johnson) is another lens with similar features. Iris-claw lenses are fixed to the midperiphery of the iris and do not need the support of the angle or ciliary sulcus and hence do not interfere with normal anatomical structures. Due to its vaulted structure, it has the advantage of decreasing the risk of pupillary blockade. Iris-claw lenses can be placed in anterior or posterior to iris tissue. Mora et al. in their retrospective comparative study found comparable safety and functional outcomes between the anterior vs. retropupillary iris-claw groups. [24] Forlini et al. published a retrospective analysis of long-term follow-up of retropupillary ICIOL implantation in 320 patients and concluded that complications related to retropupillary iris-claw were minimal compared with its benefits [25]. This technique has an easy learning curve, short surgical time, and low incidence of perioperative complications. Complications include large corneal incision, iritis, cystoid macular edema, raised intraocular pressure, and irregular shape of pupil. We found the comparable rate of complications when compared to other studies [10, 26, 27].

A single patient had an iris-claw drop, which required a secondary surgical procedure for reenclavation. The slow visual recovery related to high postop astigmatism and slow refraction stability hampers a correct postop lens prescription, creating a long discomforting period of low visual acuity. Astigmatic stabilization can occur even after 6 months, as previously described by Chen et al. [28]. This makes the iris-claw lens relatively less desirable owing to reduced patient's satisfaction and prolonged visual recovery time. Moreover, the pupil deformation risk related to a wrong enclavation procedure could be responsible for the patient's dysphotopic phenomenon [10, 27]. To avoid complications related to abnormal pupillary shape, a newer surgical technique using a guide needle to facilitate exact and equidistant enclavation has been tried [29].

Carlevale et al. in 2020 introduced a new type of lens (SOLEKO) [12, 13]. This lens is provided by a small harpoon suited for the sutureless lens anchorage to the sclera by a 23G sclerotomy protected by a scleral flap. Carlevale's IOL is a hydrophilic one-piece IOL with a 6.5 mm optic plate and a wide diameter of 13.5 mm. This allows the use of the previous phacoincision along with the minimally invasive injection technique for IOL insertion (medical Viscoject 2.2 mm). The advantage is the possibility of a rapid visual recovery with less induced astigmatism. Complications such as vitreous hemorrhage can occur, which was seen in one of our patients. Lens injection maneuver requires skill and caution in a dilated pupil with the absence of capsular support. There is a chance of subluxation of the IOL in the vitreous cavity. This complication did not occur in our cohort of study.

All three surgical procedures for secondary IOL implantation showed similar functional recovery without statistically significant differences (*p* > 0.05). The Carlevale's IOL group showed higher postop corrected visual acuity, although this was not statistically significant (*p* > 0.05). A longer follow-up period may possibly capture some complications not manifested during the study period.

## 5. Conclusions

All procedures resulted in good visual outcome in the included cohort. The associated complications were infrequent, treatable, and not related to visual acuity. Relatively small study population was one of the limitations of this study.

We feel that a randomized trial with a higher number of subjects and a longer follow-up period may possibly confirm our findings.

## Figures and Tables

**Table 1:** Causes of capsular inadequate support and types of IOL implanted.

	Eyes (*n*)	Percentage (%)	Iris-claw	Intrascleral fixation	Carlevale's lens
Posterior capsular rent (PCR)	10	41	6	4	0
Subluxation secondary to PXF	6	25	3	0	3
Subluxation secondary to Marfan's syndrome	3	12.5	2	0	1
IOL subluxation	3	12.5	1	2	0
Traumatic cataract	2	9	1	0	1
Total	24	100	13	6	5

**Table 2:** Primary or secondary type of surgery.

	Eyes (*n*)	Primary surgery				Secondary surgery	
		PHACO	FLACS	ICCE	Subluxation secondary to PXF	Anterior vitrectomy	Posterior vitrectomy
PCR	10	10	0	0	0	4	6
PXF subluxation	6	5	0	1	0	3	3
Marfan's subluxation	3	2	1	0	0	3	0
IOL subluxation	3	0	0	0	3	0	3
Traumatic cataract	2	2	0	0	0	0	2
Total	24	19	1	1	3	10	14

**Table 3:** Mean preop and postop mean visual acuity.

BCVA	Preop	Postop	*P* value
logMAR	0.49 ± 0.2	0.19 ± 0.1	<0.0001

**Table 4:** Intergroup postop visual acuity results of ANOVA and Bonferroni test.

Postoperative BCVA	Carnevale vs. iris-claw	Carnevale vs. transscleral	Iris vs. transscleral	ANOVA
(logMAR) *p* values	0.672	1	1	0.458

**Table 5:** Incidence of complications related to the lens implanted.

	Eyes (*n*)	Percentage	Iris-claw	Transscleral	Carlevale
Raised IOP	4	16	2	2	0
IOL malposition	2	8	1	1	0
Corneal complications	4	16	2	2	0
CME	1	4	1	0	0
RD	0	0	0	0	0
Pupillary anomalies	3	12.5	3	0	0
VH	2	8	0	1	1
High astigmatism	7	29	7	0	0

## Data Availability

The data used to support the findings of this study are available from the corresponding author upon request.

## Supplementary Materials

Supplementary Materials Video 1. Surgical technique of anterior iris-claw fixation. Video 2. Carlevale technique of IOL fixation.
