# Supplementary material for: Supporting IOL'S in a Deficient Capsular Environment: The Tale of No “Tails”
Source: J Ophthalmol. 2021 Sep 13;2021:9933486. doi: 10.1155/2021/9933486 (PMC8455193; doi:10.1155/2021/9933486)
Supplement: Supplementary Materials — Video 1. Surgical technique of anterior iris-claw fixation. Video 2. Carlevale technique of IOL fixation. [file 9933486.f1.docx]

**Supplementary Material-**

Video 1 Describes surgical technique of anterior iris claw fixation.

(Supplementary Video 1-https://drive.google.com/file/d/1a6SridVD9ZBNOmdvftMtTeK-NH3-nf7Y/view?usp=sharing)

Video 2 Demonstrates Carlevale technique of IOL fixation.

(Supplementary video 2- <https://drive.google.com/file/d/1uR_B8y4j0Nwi3LerWOMcoCmwGam7-ktU/view?usp=sharing>).
